# Increasing impacts of fire air pollution on public and ecosystem health

**DOI:** 10.1016/j.xinn.2024.100609

**Published:** 2024-03-05

**Authors:** Xu Yue, Yihan Hu, Chenguang Tian, Rongbin Xu, Wenhua Yu, Yuming Guo

**Affiliations:** 1 Jiangsu Key Laboratory of Atmospheric Environment Monitoring and Pollution Control, Collaborative Innovation Center of Atmospheric Environment and Equipment Technology, Nanjing 210044, China; 2 School of Environmental Science and Engineering, Nanjing University of Information Science & Technology, Nanjing 210044, China; 3 Climate, Air Quality Research Unit, School of Public Health and Preventive Medicine, Monash University, Melbourne, VIC 3004, Australia

## Main text

**Figure 1:**
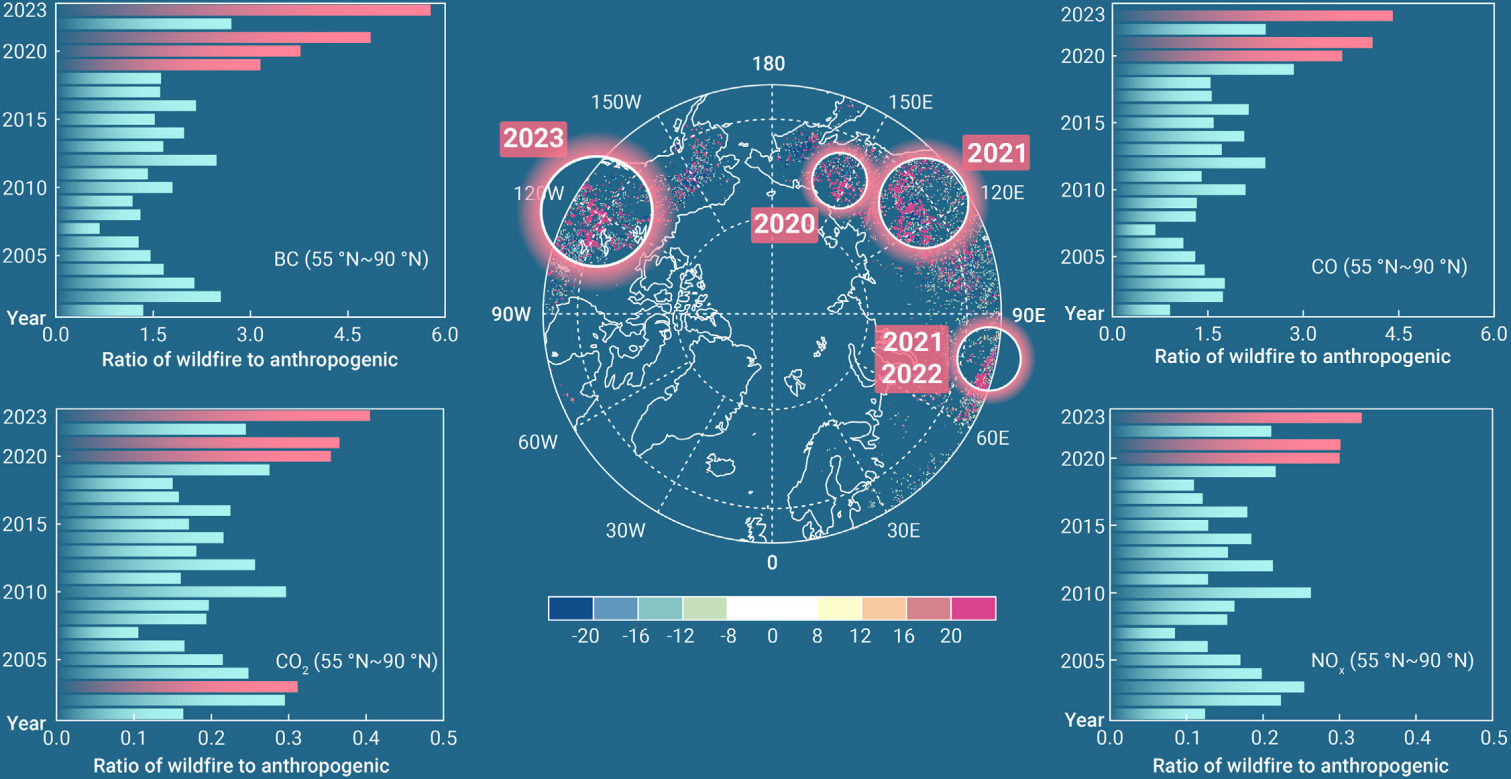
Fire emissions in boreal regions The map shows the difference in fire carbon dioxide (CO_2_) emissions (unit: g C m^−2^ year^−1^) north of 55°N between the averages of 2019–2023 and 2001–2018. Red circles indicate significant combustions in the specific years. The bars indicate the year-to-year ratios of black carbon (BC), CO_2_, carbon monoxide (CO), and nitrogen oxides (NO_x_) between wildfire and anthropogenic emissions summed for northern 55°N from 2001 to 2023. Ratios greater than 3 for BC or CO and 0.3 for CO_2_ or NO_x_ are marked in red. The Quick Fire Emissions Dataset (https://portal.nccs.nasa.gov/datashare/iesa/aerosol/emissions/QFED/v2.6r1/0.1/QFED/) is used for fire emissions. The annual total emissions are calculated except that the first 9 months of data are used for 2023. Historical anthropogenic emission datasets are adopted from the Coupled Model Intercomparison Project phase 6 with the updated COVID-19 impacts for 2020–2022 (https://esgf-node.llnl.gov/search/input4mips/).

Wildfire episodes have become more frequent and severe in recent years.^1^ Record-breaking fires devastated the Arctic, Amazon, and Australia in 2019–2020. This year, fires began in Canada in May and lasted for several months, resulting in an area burned of 16.5 million hectares by early September. This size is 6–7 times the annual fire area for a normal year in Canada. The favorable fire weather for burning and spread lasted for months (https://cwfis.cfs.nrcan.gc.ca/maps/fw). Furthermore, most Canadian fires occur in remote regions far from firefighting facilities, causing fire extinction to be difficult. Unfortunately, such “unprecedented” fire events occurred routinely in boreal regions between 2020 and 2023, although the locations varied from year to year (Figure 1).

Biomass burning releases tremendous amounts of CO_2_ into the atmosphere, contributing to global warming. Moreover, fire emissions generate many air pollutants (see the fire inventory link in the legend for Figure 1), including but not limited to particulate matter (PM), such as black carbon (BC), and trace gases, such as carbon monoxide (CO) and nitrogen oxides (NO_x_). The fire-emitted gas precursors can further produce ozone (O_3_), which is a secondary air pollutant generated through photochemical reactions between NO_x_ and volatile organic compounds (VOCs). Fire emissions usually generate a large amount of NO_x_ that can react with the abundant biogenic VOCs in forests and promote O_3_ pollution. The amount of greenhouse gases and air pollutants generated from fire emissions depends on the amount of biomass burned. For the same area, the burning of forests usually generates more emissions than the burning of grassland or cropland due to the larger fuel load of trees. On the global scale, fire emissions have remained stable over the past two decades,^2^ although the total area burned has declined, especially over central Africa. This inconsistency is caused by the contrasting changes in increasing forest fires and decreasing savanna fires.^2^ The increased fire emissions from boreal forests (Figure 1) offset the decline in emissions in tropical grasslands, leading to an increase in fire emission intensity per unit of area burned.^2^

Moreover, fire emissions worsen the air quality in downwind regions. For example, fire plumes from Canada were transported to the US and increased the concentrations of PM_2.5_ in New York to 150 μg m^−3^ on June 6^th^, which was nearly 25 times the average daily PM_2.5_ level.^1^ In addition to these megacities, fire emissions on average increased the PM_2.5_ concentration by 1–6 μg m^−3^ over boreal regions between 2000 and 2019.^3^ For these pristine regions, fire emissions were usually the major sources of regional air pollutants. As revealed by satellite retrievals, the ratios between fire and anthropogenic emissions were greater than 1 for both BC and CO in most years north of 55°N (Figure 1). These ratios showed an upward trend in the past 2 decades and exceeded a factor of 3 for the first time between 2019 and 2023. The rapid increase in fire emissions reversed the recent trends of surface PM_2.5_ in nearly three-quarters of the states in the contiguous US,^4^ where PM_2.5_ has been decreasing steadily since 2000 with anthropogenic emission control following the Clean Air Act. Furthermore, the CO_2_ and NO_x_ emitted from boreal fires accounted for more than 30% of the anthropogenic sources in three of the four recent years (Figure 1), indicating that fires are escalating global warming tendencies by generating more greenhouse gases (CO_2_ and O_3_).

Air pollutants from fire emissions are detrimental to public health. PM_2.5_ can be inhaled deep into the human body due to its small size. Furthermore, the aging and oxidative processes of chemical components during long-range transport may promote the toxicity of fire aerosols, leading to the exacerbation of respiratory and cardiovascular conditions and increased rates of mortality.^1^ On the global scale, population exposure to fire air pollutants increased, especially for PM_2.5_, between 2000 and 2019,^3^ following the positive trends of fire emissions. However, such exposure was unevenly distributed because of the spatial heterogeneity of global wildfire events. Anthropogenically driven fires at low latitudes result in greater population exposure to fire air pollutants over central Africa. In comparison, fire emissions at high latitudes exert much smaller impacts on public health, although some episodes, such as the Canadian fires, caused spikes in air pollutants^1^ because most boreal fires spread in remote areas far from residences. On average, the concentrations of fire PM_2.5_ and O_3_ were approximately four times greater in low-income countries than in high-income countries.^3^ Such socioeconomic disparities reveal the considerable health risks caused by fire activities in less-developed countries, which is an essential aspect of climate injustice.

Apart from public health risks, the impact of fire air pollution on ecosystem functions is also an urgent issue that requires more public and research attention. Aerosols can increase diffuse radiation, which is more beneficial for plant photosynthesis, by illuminating leaves under shaded conditions or in the deep canopy. This aerosol diffuse fertilization effect is more effective in boreal regions with relatively low cloud cover and large solar zenith angles, the latter of which result in a high percentage of the shading fraction for the tree canopy.^5^ In contrast, fire O_3_ tends to reduce plant photosynthesis due to its damaging effects on leaf membranes and cells. Globally, fire O_3_ has higher concentrations in tropical areas due to greater emissions of NO_x_ and more favorable climatic conditions. As a result, fire O_3_ induces a loss of gross primary productivity (GPP) up to 2% in central Africa and 3.6% in Indonesia.^5^ The joint effects of fire air pollutants are dominated by the negative impacts of fire O_3_, leading to a reduction of −0.6% year^−1^ in global GPP between 2002 and 2011.^5^ This GPP loss was much greater than that of −0.1% year^−1^ induced by drought between 2000 and 2009, suggesting the nonnegligible effects of fire air pollution on ecosystem functions.^5^

Increasing fire emissions pose an urgent need to examine the impacts of fire air pollutants on public and ecosystem health. Such studies require advancements in technology to isolate fire air pollutants from other sources. Chemical transport models (CTMs) are useful tools for quantifying the contributions of fire emissions to atmospheric components by comparing the differences between simulations with and without fire perturbations. However, the uncertainties in emission inventories, meteorological forcings, and chemical mechanisms jointly cause biases in the simulated concentrations of PM_2.5_ and O_3_ relative to the observed concentrations.^3^ The combination of machine learning (ML) algorithms helps enhance the predictivity of CTMs and derives the most accurate estimates of both all-source and fire-sourced air pollutants.^3^ In addition, ML algorithms could downscale the predictions from the originally coarse resolutions to much finer grids with relatively low computational costs. The joint application of CTMs and ML algorithms provides a powerful tool for distinguishing the quota of fire emissions in total air pollutants. As an alternative, satellite images are used to identify all ground stations that fall within fire smoke areas on specific days.^4^ Then, the fire-induced perturbations to surface PM_2.5_ are calculated as the PM_2.5_ anomalies of these smoke days deviating from the median values of recent months and nearby locations on nonsmoke days.^4^ It is encouraging that the CTM-ML and satellite-ground approaches showed good consistency in terms of the derived fire PM_2.5_ in the US,^3^ which provides independent support for both methods. The application of satellite-ground approaches is limited to regions (e.g., the U.S.) with dense coverage of ground stations and daily monitoring of fire plumes. In comparison, the CTM-ML approach has several advantages in deriving concentrations of fire air pollutants at the global scale and over regions without satellite-based smoke polygon products. Moreover, given that the CTM could separate different emission sources, the CTM-ML approach may isolate air pollutants caused by specific sources other than fire emissions.

With these advancements, we expect to extend the assessment of the impacts of fire air pollution on populations and ecosystems. Such explorations include but are not limited to (1) establishing links between morbidity/mortality for specific diseases and fire plume exposure globally, particularly in less developed countries. (2) comparing public health risks between fire-emitted and anthropogenic-driven air pollution. (3) distinguishing biospheric responses to fire air pollutants for various ecosystem types. and (4) projecting future fire emission impacts under different climate change scenarios. Given the increasing contributions of fire emissions to regional and global air pollution, more efforts should be made to explore protection and mitigation strategies for maintaining public health and ecosystem functions.
